# Micro-patterned agarose gel devices for single-cell high-throughput microscopy of *E. coli* cells

**DOI:** 10.1038/s41598-017-17544-2

**Published:** 2017-12-21

**Authors:** David G. Priest, Nobuyuki Tanaka, Yo Tanaka, Yuichi Taniguchi

**Affiliations:** 1 Laboratory for Single Cell Gene Dynamics, Quantitative Biology Center (QBiC), RIKEN, 6-2-3 Furuedai, Suita, Osaka 565-0874 Japan; 2 Laboratory for Integrated Biodevice, Quantitative Biology Center (QBiC), RIKEN, 1-3 Yamadaoka, Suita, Osaka 565-0871 Japan; 3 PRESTO, Japan Science and Technology Agency, 4-1-8 Honcho, Kawaguchi, Saitama 332-0012 Japan

## Abstract

High-throughput microscopy of bacterial cells elucidated fundamental cellular processes including cellular heterogeneity and cell division homeostasis. Polydimethylsiloxane (PDMS)-based microfluidic devices provide advantages including precise positioning of cells and throughput, however device fabrication is time-consuming and requires specialised skills. Agarose pads are a popular alternative, however cells often clump together, which hinders single cell quantitation. Here, we imprint agarose pads with micro-patterned ‘capsules’, to trap individual cells and ‘lines’, to direct cellular growth outwards in a straight line. We implement this micro-patterning into multi-pad devices called CapsuleHotel and LineHotel for high-throughput imaging. CapsuleHotel provides ~65,000 capsule structures per mm^2^ that isolate individual *Escherichia coli* cells. In contrast, LineHotel provides ~300 line structures per mm that direct growth of micro-colonies. With CapsuleHotel, a quantitative single cell dataset of ~10,000 cells across 24 samples can be acquired and analysed in under 1 hour. LineHotel allows tracking growth of > 10 micro-colonies across 24 samples simultaneously for up to 4 generations. These easy-to-use devices can be provided in kit format, and will accelerate discoveries in diverse fields ranging from microbiology to systems and synthetic biology.

## Introduction

High-throughput microscopy of bacterial cells has yielded new insights in molecular biology such as genome-wide characterisation of protein localisation^1^ and gene expression noise^2^. Acquiring high-throughput datasets containing multiple images of hundreds of different samples (strains or conditions) requires development of multi-sample imaging devices. Also, since bacterial cells, such as the rod-shaped *E. coli*, are small (approximately 1 by 3 μm), in order to acquire quantitative images, cells must be held still and flat under the microscope. PDMS microfluidic devices have been developed for high-throughput imaging using microfabrication techniques to generate microfluidic channels or chambers^2,3^. PDMS microfluidic devices are highly customisable, however drawbacks include the time and expertise required to produce each device. For example, while our previous PDMS device could efficiently image 96 samples, punching the 96 fluidic inlets and outlets in the device is time consuming and requires careful attention^2^. We therefore sought an easy-to-use device to implement high throughput imaging.

The agarose gel pad is an alternative technique where cells are physically sandwiched between a thin (~1 mm), flat agarose gel pad and the coverslip^4–6^. Agarose gel is soft and gentle to cells, and can be made containing growth medium for live-cell, time-lapse imaging. Recently, large-format agarose pads have been used for high-throughput imaging^1,7^. However these devices suffer from two main drawbacks. Firstly, adding multiple samples as liquid droplets to one agarose pad results in cross-contamination if droplet size is not kept to a minimum (~2 μL). Secondly, cells have a tendency to aggregate into clumps on the flat gel surface. Images of densely packed or clumped cells are difficult to segment during image analysis, and require time-consuming manual curation to remove mis-detected cells.

An ideal device for high-throughput imaging would combine the customizability of PDMS, with the ease-of-use and beneficial characteristics of agarose gel. A previous study found that like PDMS, the surface of agarose gel could be imprinted with micro-patterned features^8^. Their device employed thin parallel ‘tracks’ (approximately equal to the cell width), that forced cells to grow outwards in a straight line instead growing into clumped micro-colonies. The tracks terminated in larger ‘gutters’ where fluid flow removed excess cells to permit long term imaging. Cells growing in a straight line are easier to analyse since individual cell boundaries are more readily detected during image analysis. A drawback of this device however is that it can only measure one sample at a time, and so cannot be used for high-throughput imaging.

Here, we present two new micro-patterned agarose devices called CapsuleHotel and LineHotel, for high-throughput imaging of *E. coli* cells. Each device incorporates 24 micro-patterned agarose pads tailored for single-shot imaging of thousands of cells (CapsuleHotel) or tracking of micro-colony growth (LineHotel). CapsuleHotel employs a new micro-patterned agarose design, a grid of cell-sized ‘capsules’, that trap individual *E. coli* cells and prevent cell clumping to allow fully automated analysis of high-throughput single cell datasets. To demonstrate the capability of CapsuleHotel, we obtain reproducible, quantitative fluorescent reporter gene noise measurements on a set of strains from an *E. coli* Venus-tagged library^2^. LineHotel uses track (or ‘line’) micro-patterns on each pad without fluid flow to allow straightforward tracking of growing cells for up to four generations across 24 samples simultaneously. These new devices are easy-to-use, do not require any microfabrication equipment, and can be provided in ‘kit’ form to any lab with a computer controlled microscope.

## Results

### Multi-pad agarose gel pad for high-throughput bacterial microscopy

**Figure 1:**
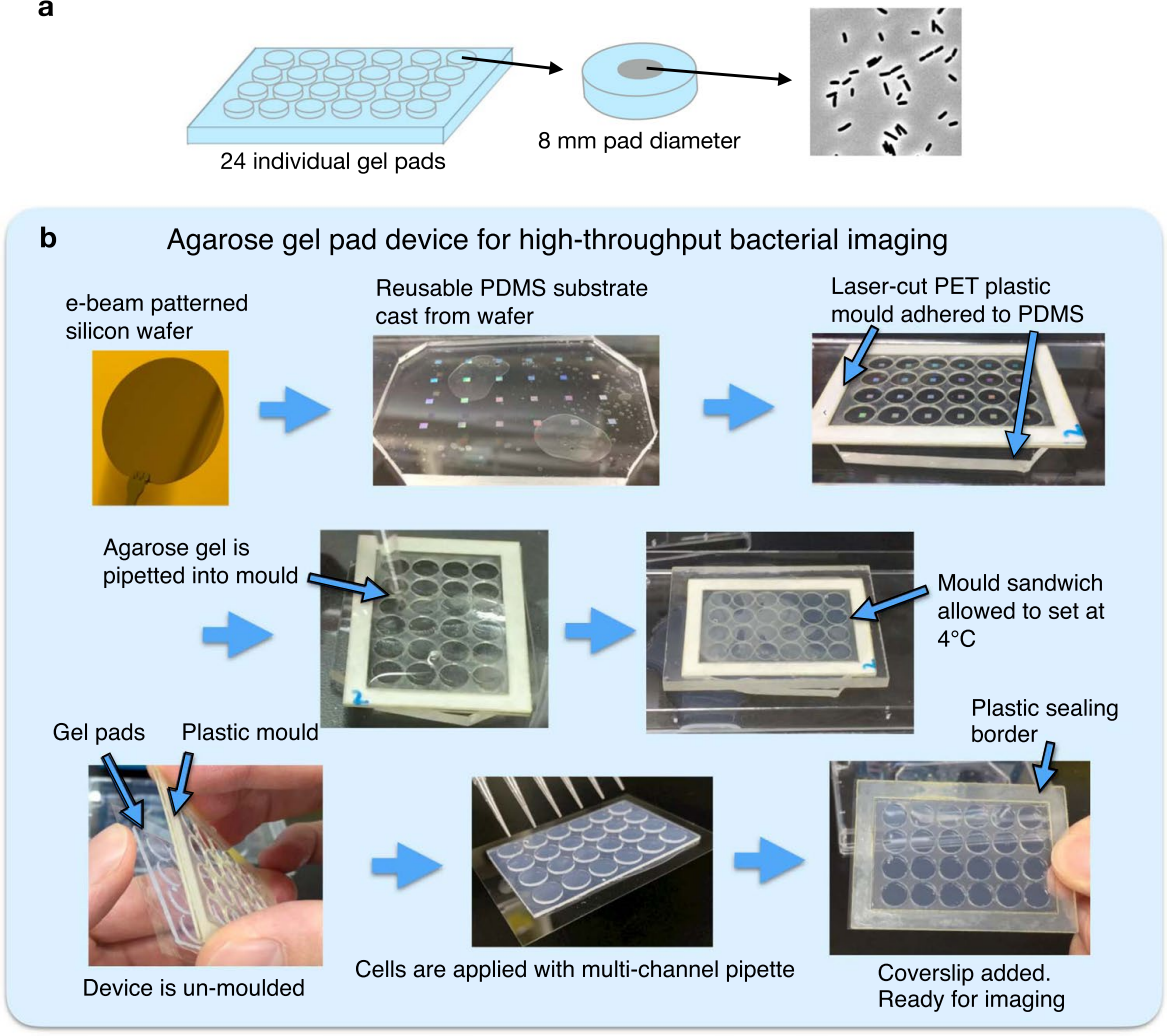
Multi-pad agarose gel pad device provides an easy-to-use platform for high-throughput bacterial microscopy. (**a**) Multi-pad agarose gel pad device allows a separate sample to be added to each pad for high-throughput imaging. (**b**) Steps in making micro-patterned multi-pad agarose gel pads. From top left: Electron beam lithography is used to generate micro-patterning on a 3 inch silicon wafer. PDMS is cast onto the wafer. Laser cut PET plastic is adhered to the PDMS using double-sided tape. Molten agarose is poured into the mould and set at 4 °C. The gel is un-moulded and cells are pipetted onto each pad. After the liquid has dried, a coverslip is added and the device is sealed and ready for imaging.

We aimed to create an agarose gel pad-based device for high-throughput imaging of bacterial cells. Previous high-throughput agarose gel devices used large, flat agarose pads (50 by 70 mm or larger)^1,7^, however since multiple samples are added the same pad, cross-contamination can occur between adjacent liquid droplets. To avoid this problem, we created a 6 by 4 array of 8 mm round gel pads spaced to fit a multi-channel pipette by adhering custom plastic moulds with double-sided tape to a PDMS sheet (Fig. 1, see Methods). The tightly-controlled gel thickness allows use of a custom plastic border to seal the device, preventing gel shrinkage during imaging and associated XY drift.

Throughout the paper, to test our devices, we imaged strains from an *E. coli* genomic C-terminal Venus-tagged library^2^. A set of strains from the library were subcultured from an overnight culture and grown to early log phase (OD_600_ = 0.1–0.4) in a microtitre plate, washed in PBS and pipetted onto each pad of the multi-pad device (5 μL of cells at OD_600_ = 0.2–0.8). Liquid droplets on the gel pads were allowed to dry before adding the coverslip and commencing imaging. Nine phase contrast and fluorescence images per pad were acquired automatically using an inverted microscope with motorised stage. The gaps between each pad in the device prevented unwanted merging of liquid drops and thereby eliminated cross contamination between samples. On this multi-pad device, we found that cells on flat gel pads often aggregated into massive clumps, which could not be analysed automatically during image analysis (Figure S1, S3).

### CapsuleHotel for high-throughput, single-shot imaging

**Figure 2:**
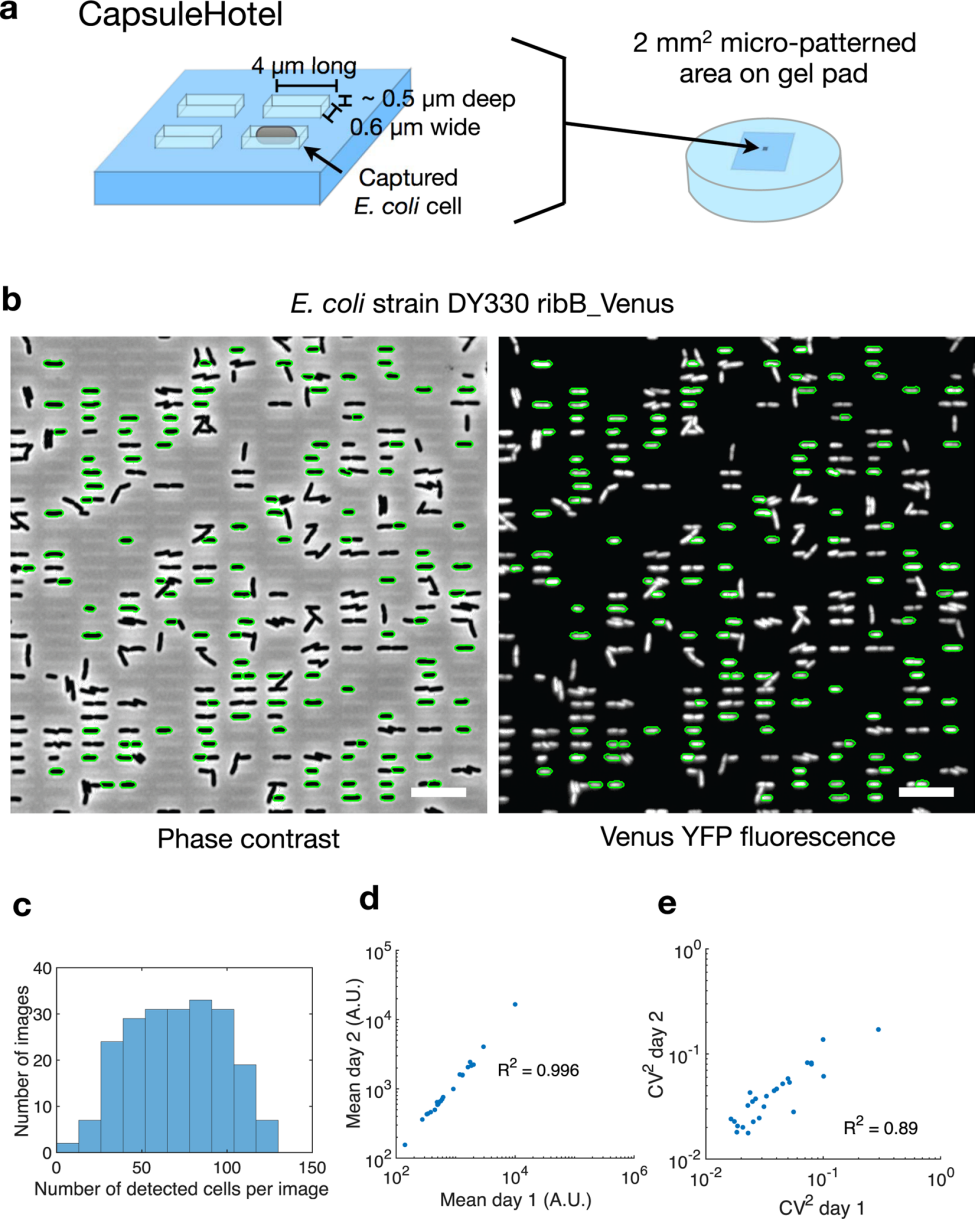
CapsuleHotel micro-patterned multi-pad agarose device for high-throughput imaging. (**a**) Design for a micro-patterned agarose pad to physically segregate single cells. (**b**) Cells applied to capsule micro-patterned agarose pad are physically segregated. Phase contrast (left) and YFP fluorescence (right) images of DY330 ribB_Venus are shown. Automatically detected cells are circled in green. Scale bar = 10 μm. (**c**) A set of DY330 Venus library strains automatically imaged across this device yielded an average of 70 cells per image from 207 images. (**d**,**e**) A set of DY330 Venus library strains imaged on two separate days gave reproducible fluorescence mean (**d**) and noise (**e**). See also Table S1.

To address the clumping issue, inspired by previous findings on micro-patterned agarose^8^, we created CapsuleHotel, a micro-patterned multi-pad agarose gel device for high-throughput single-shot imaging. We reasoned that a grid of cell-sized rectangular boxes (‘capsules’) (each sized 4 μm long by 0.6 μm wide by ~0.5 μm deep) would trap individual cells and prevent aggregation into clumps (Fig. 2a). We developed a new electron beam lithography protocol for etching user-defined micro-patterns onto a 3-inch silicon wafer (see methods). Micro-patterned designs were then transferred to a PDMS intermediate, which served as a master onto which agarose gel pads were repeatedly cast (Fig. 1b).

Cells prepared as above were pipetted onto the pads of CapsuleHotel, and indeed, the capsule patterning robustly captured individual cells across all the pads (Fig. 2b). A typical imaging session yielded phase contrast and fluorescence data for 14998 cells across 207 images at an average of 70 cells per imaging frame (image size = 85 μm^2^) in less than 20 minutes (Fig. 2c). Due to the softness of agarose gel, a single capsule size effectively captured cells of varying sizes (Figure S2). We further found that the capsule pattern maintained single cell isolation at high cell densities (OD_600_ = 5 as added to the device) that always result in clumping on non-patterned gel pads (Figure S3). To demonstrate reproducible high-throughput measurements with CapsuleHotel, we imaged a set of Venus-tagged library strains on two days and compared their fluorescence mean (Fig. 2d) and noise (the coefficient of variation squared (CV^2^)) (Fig. 2e, Table S1). The mean fluorescence (R^2^ = 0.996) and noise (R^2^ = 0.89) were highly reproducible from day to day.

### LineHotel for high-throughput, time-lapse imaging

**Figure 3:**
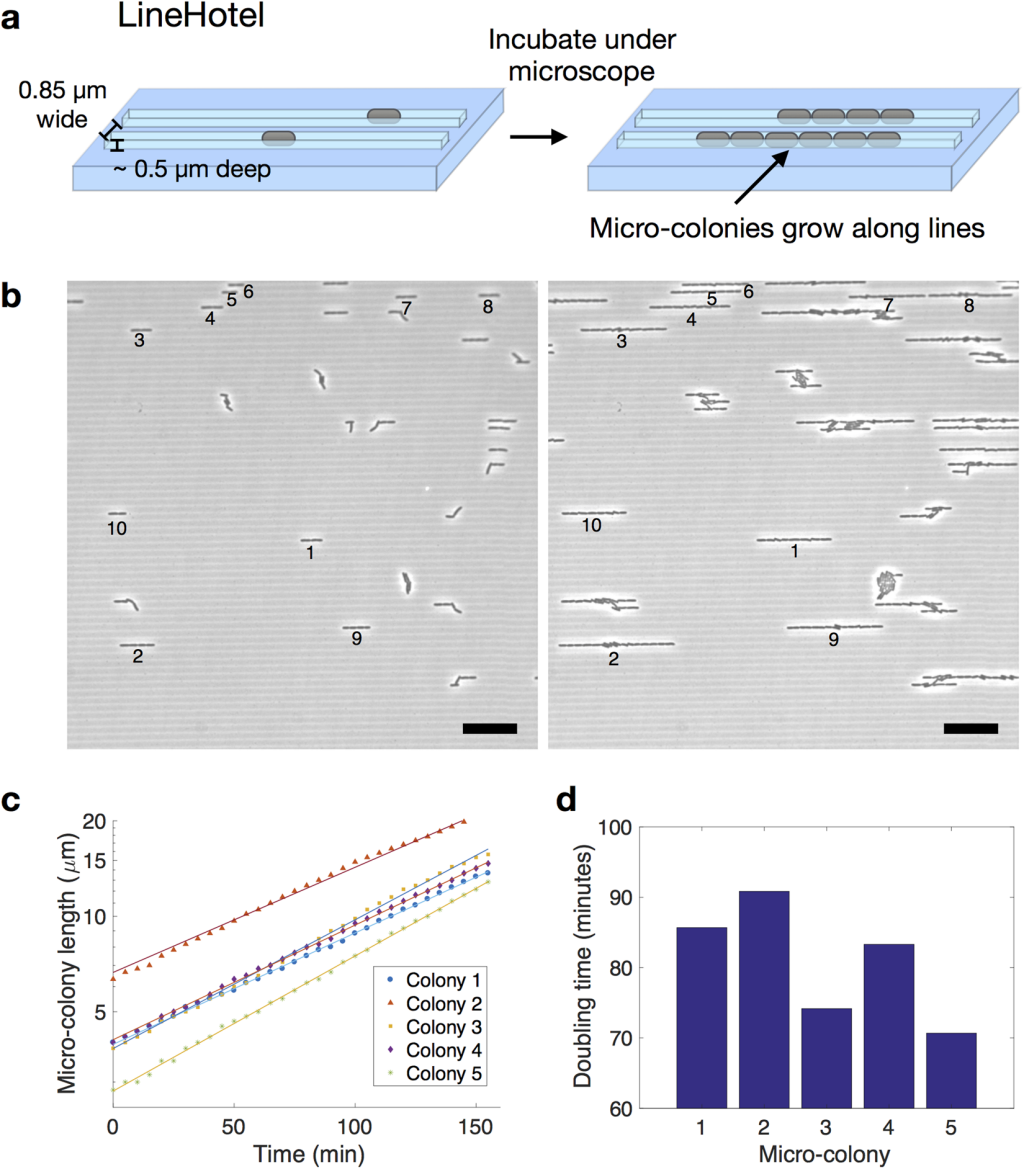
LineHotel for tracking growing micro-colonies (**a**) Design for a micro-patterned agarose pad to direct micro-colony growth in straight line. Hundreds of parallel lines (0.85 μm width, 2 μm spacing) are patterned onto the agarose surface. (**b**) *E. coli* cells from an exponentially growing culture were pipetted onto 24 separate pads of LineHotel and imaged as a time-lapse at 30 °C. Phase contrast images from one pad are shown at 0 (left) and 155 (right) minute timepoints. Analysed micro-colonies are numbered 1–10. Scale bar = 10 μm. (**c**) The length of 5 micro-colonies (from b) vs time was obtained from the phase contrast images and fit with an exponential growth curve. (**d**) Doubling times for micro-colonies from c.

Single-shot imaging using CapsuleHotel can obtain a snapshot of states across populations of cells, however experiments often require dynamic, time-lapse information from growing micro-colonies^6,9^. Therefore, to track live cell growth, we used our e-beam technique to create LineHotel, a multi-pad device with the ‘line’ pattern (line width 0.85 μm) (Fig. 3a). Cells from an exponentially growing culture were diluted and pipetted directly onto the 24 pads of LineHotel (5 μL at OD_600_ ~ 0.05) and imaged as a time-lapse in an incubated microscope (Fig. 3b). Growth of single micro-colonies was quantitated by measuring the rate at which the length of the line of cells expanded (Fig. 3c,d). Micro-colonies grew exponentially at different rates for 4 cell generations (from 1 to approximately 16 cells long) after which cells started growing out of the tracks (Fig. 3b).

## Discussion

Here, we have created a micro-patterned agarose platform for high-throughput quantitative bacterial microscopy. Firstly, with CapsuleHotel, we used a grid of micro-capsules that trap individual *E. coli* cells to allow fully automated, high-throughput imaging (Fig. 2). Secondly, we created LineHotel for live-cell time-lapse imaging of growing micro-colonies across 24 different samples (Fig. 3).

CapsuleHotel physically segregates *E. coli* cells from a wide range of culture density, thereby avoiding the need to manually normalise the cell concentration of liquid cultures (Figure S3). Also, the physical segregation of single cells avoids any fluorescence bleed-through that can occur between touching cells. Device preparation (Fig. 1) is quicker and simpler than disposable PDMS devices, which require PDMS degassing and curing, plasma sealing to coverslips, and preparation of microfluidic inlets and outlets. It takes 30 minutes to prepare four 24-pad CapsuleHotel devices (96 samples) with cells for imaging and a further 20 minutes to image each device on a newer microscope with hardware focus. This ease-of-use combined with high throughput is useful where multiple strains need to be quickly characterised, such as quantitation of fluorescent spots^10–12^ and gene expression noise^2,6^.

LineHotel can be used to track micro-colony growth across 24 samples for 4 generations, which is sufficient to estimate the single-colony growth rate, and correlate it with other measurements, such as cellular fluorescence. Beyond 4 generations, cells begin to grow out of the lines, however we found that if wider lines are used (>0.9 μm), cells move up and down the lines, which hinders growth quantitation. Therefore, in order to track growth for longer than 4 generations, a more complicated two-layer device incorporating fluid-flow to remove daughter cells would be required. Further development of micro-patterned agarose combined with a PDMS enclosure is a promising candidate for such a device^8^.

Fabricating the micro-patterned silicon wafer requires an e-beam facility, however multiple PDMS moulds can be cast from each wafer and at least 100 agarose gels can be cast from each PDMS mould. An epoxy master can also be cast from the PDMS moulds for long-term storage. Throughput could be increased to 96 pads per device by placing four 24-pad gels onto a larger coverslip, however this would require a custom microscope sample holder. In principle, throughput could also be increased by decreasing the gel pad size, however we found that decreasing the pad size below 8 mm caused a range of problems such as unavoidable air bubbles in the smaller gel pads. Our e-beam protocol allows arbitrary two-dimensional patterns to be drawn onto a 3-inch silicon wafer at a feature resolution of approximately 50 nm, opening the door to diverse cell types and experimental designs.

Here, by combining a micro-patterned PDMS substrate and laser-cut PET plastic moulds, we have created easy-to-use and robust devices for high-throughput imaging of bacterial cells. We envisage CapsuleHotel, LineHotel and other micro-patterned agarose devices will accelerate discovery across broad fields spanning microbiology, antibiotic resistance, systems and synthetic biology.

## Methods

### *E. coli* strains and growth

The *E. coli* strains in this study are from a library of endogenous C-terminal translational fusions to Venus yellow fluorescent protein^2^. The genetic background for this library is DY330 (W3310 ΔlacU169 gal490 λcI857 Δ(*cro-bioA*))^13^. The growth media is based on M9 minimal medium (11.28 g/L M9 salts 5x (BD, Difco), 0.01 mM MnCl_2_ (Nacalai Tesque), 0.01 mM FeCl_3_ (Nacalai Tesque), 0.1 mM CaCl_2_ (Nacalai Tesque), 2 mM MgSO_4_ (Sigma-Aldrich), 0.4% w/v D-(+)-Glucose (Nacalai Tesque), 0.6 μg/mL biotin (Thermo Fisher Scientific), 1x MEM vitamins (Thermo Fisher Scientific), 1.6 g/L Complete Supplement Mixture (ForMedium) and 20 μg/mL chloramphenicol (Sigma-Aldrich)), filter sterilised, herein called M9 medium.

Library strains were picked from glycerol stock plates into 200 μL M9 medium in 96-well microtitre plates either round-bottom plates for glycerol stocks and pelleting cells (267334) (Thermo Fisher Scientific), or flat-bottom plates (351172) (Falcon Corning) for cell growth with OD_600_ measurement, and sealed with adhesive seals (FG-DM100HD) (FastGene) and grown overnight (30 °C, 100 rpm shaking) in an incubated plate shaker (BioShaker MBR-022UP or MBR-024) (Taitec). Overnight cultures were mixed with 50 μL 80% glycerol and stored at −80 °C as working glycerol stocks. To grow cells for imaging, working glycerol stock plates were thawed at 30 °C and 1 μL was inoculated into 200 μL fresh M9 medium and grown overnight (30 °C, 100 rpm shaking). The next morning, overnight cultures were subcultured 1/200 into M9 medium without chloramphenicol and grown to early log phase (OD_600_ ~0.1–0.4) measured by a microplate reader (MTP-310) (Corona electric). 100–200 μL cells were then washed by first pelleting in round-bottom plates (3000 rpm, 6 min, 8 °C) in a benchtop plate centrifuge (Allegra X-22R) (Beckman coulter), removing all but ~20 μL of the supernatant, resuspending in 200 μL ice cold PBS, pelleting again, removing the supernatant, and then resuspending in 50–100 μL PBS and storing the plate on ice until imaging. Under these growth conditions the doubling time was approximately 80–90 min.

### Preparation of micro-patterned silicon wafer and PDMS moulds

Si-wafers for replicating patterns were prepared with an electron beam lithographic technique^14^. Briefly, positive electron beam resist (ZEP520A) (Nippon Zeon, Tokyo) was coated on a 3-inch-diameter Si-wafer (p-type, mirror-finished <100> surface) (SEMITEC, Chiba) with a spin-coater (MS-A150) (Mikasa, Tokyo) spinning first at 200 rpm for 5 s and then at 800 rpm for 120 s. The electron beam resist covering over the Si-wafer was baked at 180 °C for 3 min. The electron beam resist was exposed to electron beam with 50 kV acceleration voltage and 100 pA beam current in an electron beam lithography system (ELS-7500) (Elionix, Tokyo). The exposure area was 300 × 300 μm^2^, which was equivalent to 60,000 × 60,000 dots in a designing resolution, and the exposure time was 0.25 ms/dot. Multiple exposure areas were aligned to create the 1.5 mm^2^ micro-patterned area. The electron beam resist after the exposure was treated with a developer *o*-xylene (246–00105) (Wako, Osaka) for 120 s. After the developer dried, the Si wafer was ion etched using the Bosch process with dry etching system (ELS-700) (Elionix). The plasmas of C_4_F_8_ and SF_6_ were used for the overcoating and etching processes, respectively. The powers for generating the inductive coupled plasmas of C_4_F_8_ and SF_6_ and for drawing the SF_6_ plasma were 450 and 30 W, respectively. The number of overcoating and etching processes was sixteen. PDMS and a curing agent for PDMS (Sylpot 184 W/C) (Dow Corning Toray, Tokyo) were mixed at a ratio of 10 to 1 and degassed. The PDMS mixture was poured onto the Si-wafer mold and cured at 80 °C for 3 h. The cured PDMS was peeled off from the Si-wafer and trimmed, resulting in a mould for casting micro-patterned agarose gel pads.

### Preparing agarose pad device and imaging

The micro-patterned PDMS surface was cleaned by spraying with 70% EtOH and air drying with N_2_ gas, but never wiped directly. The 1.5 mm^2^ micro-patterned areas are spaced 9 mm apart in a 6 × 4 grid to fit the tip spacing of a multichannel pipette. The mould was prepared as follows. 0.5 mm Polyethylene terephthalate (PET) was adhered with one layer of double-sided tape (No. 530 R) (Nitto Denko Corporation), and a 6 × 4 grid of 8 mm round holes were cut with a laser cutting table (Speedy 100 R) (Trotec). The settings for laser cutting were; power = 50, speed = 2, passes = 2, Air assist = ON. The white backing sheet was removed and the PET piece was adhered to the PDMS surface and pressed down to remove air bubbles. Next, a laser cut border (50 × 70 mm, 5 mm wide, 0.5 mm thick PET with two layers of double-sided tape), was adhered. 10 mL of molten 2.5% agarose (UltraPure) (Thermo Fisher Scientific) was prepared in 0.85% NaCl (Nacalai Tesque) by boiling in a microwave for ~ 1 minute and then pipetted onto the mould in excess. Low melting point agarose is not required. Immediately, while the agarose was still molten, a 60 × 80 mm 5 mm thick PET sheet was placed over to seal the mould, expelling the excess agarose. The agarose pad was then allowed to set by placing the mould onto a block incubator (BI-516C) (Astec) at 6 °C for 15 min and can be stored at 4 °C for up to a week.

The finished agarose device was unmoulded by sliding off the 5 mm PET sheet and peeling the PET + agarose pad off the micro-patterned PDMS. To the back of the gel was added a 50 × 70 mm No. 1 coverslip (0.12–0.17 mm thick) (Matsunami). The PET mould was then easily removed since the agarose gel adheres to the coverslip. Next, a 1 mm thick PET border with double-sided tape on both sides (Laser cut with power = 50, speed = 0.7, passes = 2, Air assist = ON) was adhered to the back coverslip to surround the gel but on leaving the top white backing sheet. 5 μL of washed cells on ice were then pipetted directly onto the centre of the gel pads using a multi-channel pipette (Gilson), and the device was placed on the block incubator (32 °C, 10 min) until the liquid just dried. The cell droplet must be dried otherwise cells are floating around under the coverslip. Finally, the remaining white backing sheet was removed from the border and a coverslip cleaned with a plasma cleaner (Femto) (Deiner electronic) was placed clean side down onto the agarose pads. The device was then ready for automated imaging but could be stored for a few hours without affecting the results.

### Microscopy

The microscope setup consisted of an inverted microscope (IX73) (Olympus), fitted with a motorized XY stage (Prior Scientific), Z focus drive (H122) (Prior Scientific) and EMCCD Camera (iXon3) (Andor) mounted on a light table (S-2000) (Newport). Camera settings for fluorescence imaging were 16-bit, 1 MHz with 300 EM Gain and 2.4x pre-amplifier gain. Imaging was performed with a 60x, 1.42 NA phase contrast objective lens (PlanApo N Ph3) (Olympus) and 1.6x tube lens. The illumination source was 514 nm from an argon laser (Innova 90 C) (Coherent), with 518 nm dichroic mirror and 542/27 emission filter for Venus fluorescence (Semrock). Laser intensity measured at the objective lens was 30 mW (PM100D, S121C) (Thorlabs). Exposure time 100 ms using mechanical shutter (LS2, 2 mm aperture size) (Uniblitz). Image acquisition was controlled using MetaMorph software (Molecular Devices).

For time-lapse imaging with LineHotel, agarose gels were prepared using M9 medium. Imaging was done using an inverted microscope (IX83) (Olympus) fitted with a motorised XY stage (Prior Scientific) and hardware autofocus (ZDC2) (Olympus). Phase contrast images were acquired every 5 minutes at one position per pad. Cells were prepared according to CapsuleHotel, except the subculture from the overnight culture was grown to OD_600_ ~ 0.1, then diluted 1/2 into fresh medium and 5 μL was pipetted onto LineHotel gel pads.

### Image and data analysis

Image and data analysis was performed using scripts written in Matlab software (MathWorks). Image arithmetic was performed on images converted from 16-bit to double. Fluorescence images were corrected as follows. An illumination intensity profile image was created during each imaging run by pipetting a dilute solution of rhodamine onto one of the pads. Nine images were acquired and the median was taken (‘illum’ image, see below). An offset image was created by acquiring a set of images of an empty gel pad and taking the median (‘offset’ image). Each raw fluorescence image (raw) was then corrected using the following formula with relevant Matlab functions. Corrected = (raw − offset)/(illum − offset) * max(illum − offset). The corrected fluorescence image was then converted back to 16 bit.

Phase contrast images were used for cell segmentation using a previously described method^15^. The micro-patterning simplifies cell segmentation since only objects whose major axis was within 5 degrees from horizontal were selected. As a further filter, one cell of cells touching end to end was rejected to avoid counting daughter cells that had not yet separated. The fluorescence intensity for each cell was obtained by dividing the sum of the fluorescence pixels within the cell mask by the cell area (the number of pixels in the mask). Mild filtering was applied to remove objects that were too small, too large, or that were clearly fluorescence outliers. The resulting list of cells was used to calculate the fluorescence mean, variance and noise (variance divided by squared mean). For subtraction of cellular autofluorescence, the autofluorescence is assumed to be independent of Venus fluorescence. The parental strain is imaged during each imaging run and therefore its mean and variance can be directly subtracted from that of each sample strain. Micro-colony length was quantitated in ImageJ by using Analyze Particles on selected regions of binarised phase contrast time-lapse image stacks.

### Data availability

Data is available on request.

## Electronic supplementary material

Supplementary Figures

Table S1
