# Supplementary material for: Micro-patterned agarose gel devices for single-cell high-throughput microscopy of E. coli cells
Source: Sci Rep. 2017 Dec 21;7:17750. doi: 10.1038/s41598-017-17544-2 (PMC5740163; doi:10.1038/s41598-017-17544-2)
Supplement: Supplementary file 1 — Supplementary Figures [file 41598_2017_17544_MOESM1_ESM.pdf]

Supplementary Figures

Micro-patterned agarose gel devices for single-cell high-throughput microscopy of E. coli cells

David G. Priest[a], Nobuyuki Tanaka[b], Yo Tanaka[b], Yuichi Taniguchi[a,c*]

a Laboratory for Single Cell Gene Dynamics, Quantitative Biology Center (QBiC), RIKEN, 6-2-3 Furuedai, Suita, Osaka, 565-0871, Japan.
b Laboratory for Integrated Biodevice, Quantitative Biology Center (QBiC), RIKEN, 1-3 Yamadaoka, Suita, Osaka, 565-0871, Japan.
c PRESTO, Japan Science and Technology Agency, 4-1-8 Honcho, Kawaguchi, Saitama, 332-0012, Japan.
* Corresponding author.

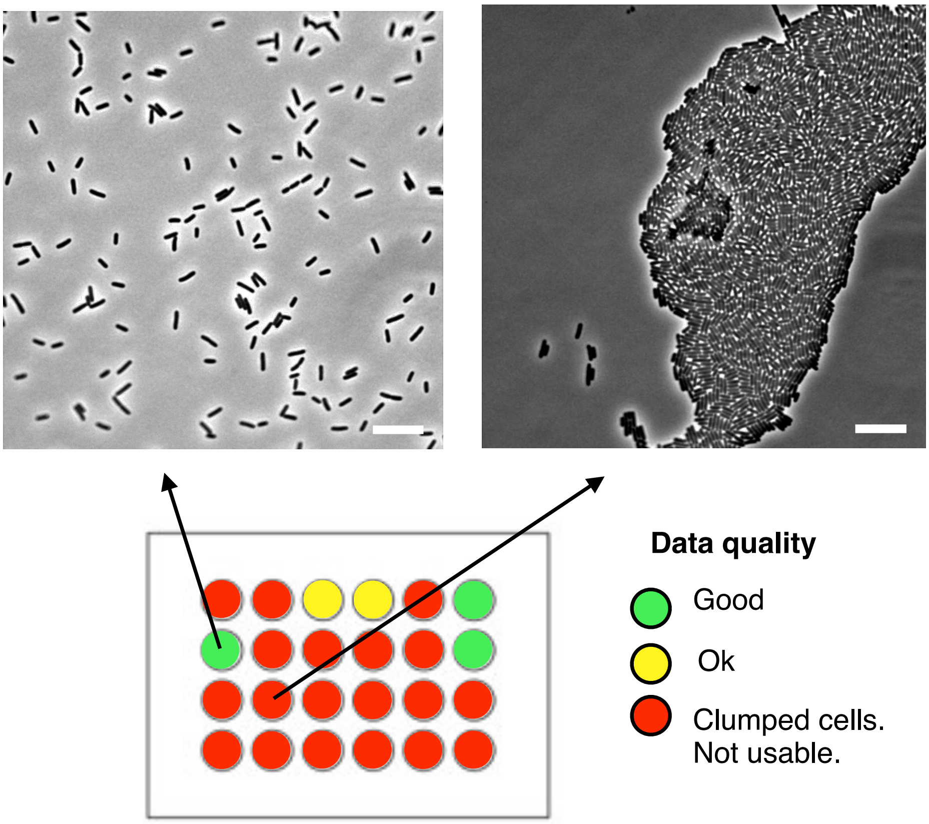

Figure S1. Cells aggregated on flat agarose gel pads, hindering high-throughput data acquisition. A 24-pad non-patterned agarose device was imaged (schematic shown below). Occasionally, well-separated cells appear on a pad (green pads, left image). Usually however, cells form large clumps as liquid droplets dry on pads (red pads, right image). Scale bar = 10 µm.

a

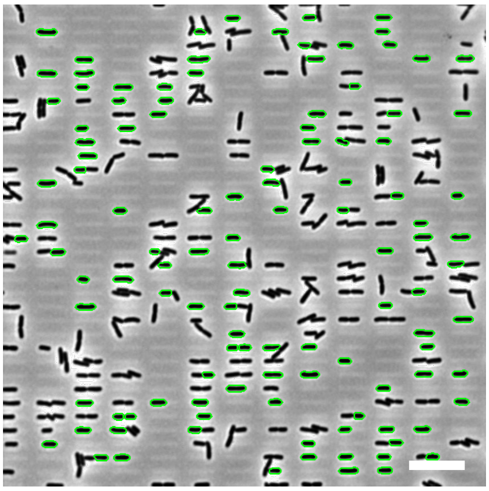

DY330 ribB_Venus
Average cell area = 109 pixels

b

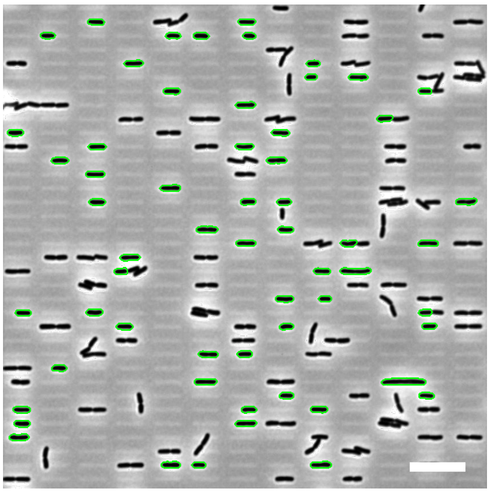

DY330 lpxD_Venus
Average cell area = 125 pixels

c

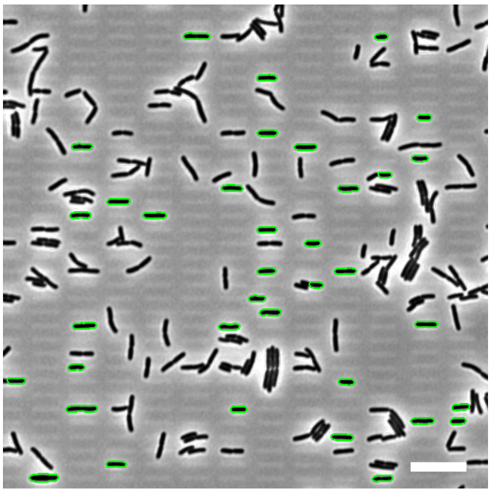

DY330 leuS_Venus
Average cell area = 141 pixels

d

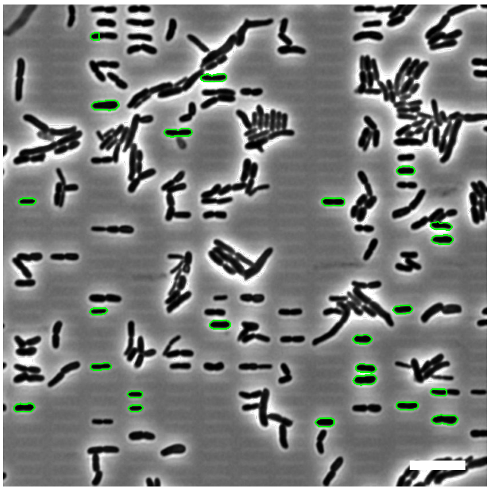

DY330 murA_Venus
Average cell area = 181 pixels

Figure S2. Micro-patterned agarose with a single capsule size (4 µm by 0.6 µm) is suitable for diverse cell sizes. Representative phase contrast images for various strains in the DY330 Venus-tagged library. a) ribB_Venus, representing smaller cells, b) lpxD_Venus, representing typically sized cells, c) leuS_Venus, larger cells and d) murA_Venus, very large and round cells. Cells were grown under the same conditions and imaged on different pads of the same multi-pad agarose device. Automatically-detected cells are circled in green. Scale bar = 10 µm.

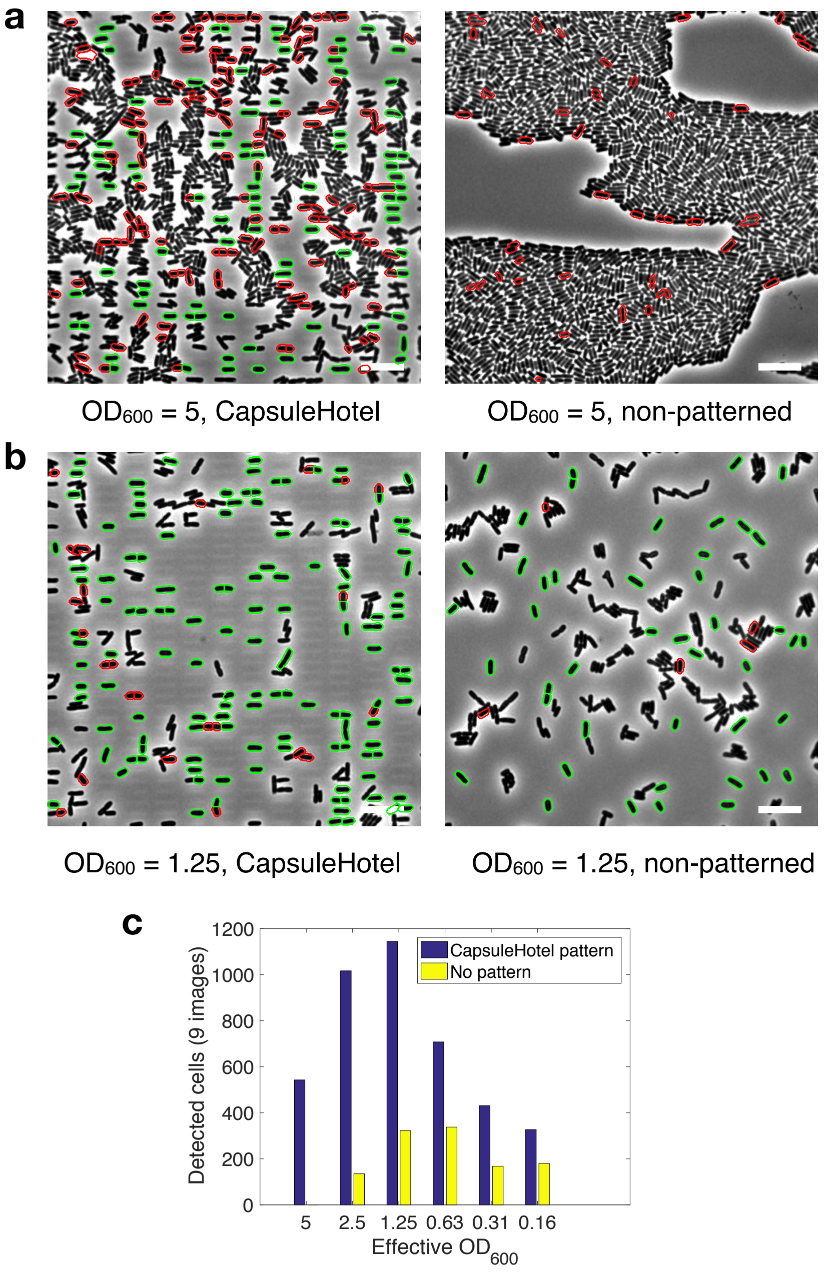

Figure S3. CapsuleHotel robustly segregates single cells over a wide range of cell densities. A culture was grown to log phase (OD600 = 0.5), washed and concentrated 10x in PBS to an effective OD600 = 5. Next 5 µL cells from a 1/2 dilution series were pipetted onto separate pads of a single CapsuleHotel device. Nine images of cells on the patterned and non-patterned area of each pad were acquired. Automatic image analysis kept cells (circled in green) if they were not surrounded by other cells, or discarded them (circled in red). a) At high density (OD600 = 5), single cells are isolated by CapsuleHotel (left), but always form clumps on non-patterned agarose (right). b) At medium and low density, CapsuleHotel robustly isolates single cells (left), whereas cells on non-patterned agarose are randomly positioned. c) Number of kept cells detected across nine images on the patterned and non-patterned area of the gel pads for the dilution series.

Table S1.
Summary data for two days imaging a set of strains from the DY330 Venus-tagged library. Each day, the strains were imaged across two multi-pad devices. Columns show: the gene name, mean cell area in pixels, mean fluorescence across the population, variance and noise (coefficient of variation squared), the autofluorescence-subtracted mean, variance and noise.
